# Use of ‘Elf Bar’ among youth and young adults who currently vape in England: cross-sectional associations with demographics, dependence indicators and reasons for use

**DOI:** 10.1111/add.16463

**Published:** 2024-03-21

**Authors:** Katherine East, Eve V. Taylor, Erikas Simonavičius, Jessica L. Reid, Robin Burkhalter, Ann McNeill, David Hammond

**Affiliations:** 1 National Addiction Centre, Institute of Psychiatry, Psychology and Neuroscience, King’s College London, London, UK; 2 School of Public Health Sciences, Faculty of Health, University of Waterloo, ON, Canada

**Keywords:** Disposables, Elf Bar, Survey, Vaping, young adults, youth

## Abstract

**Background and aims:**

Elf Bar is currently the leading e-cigarette (vape) brand in Great Britain. This study examined youth and young adults’ use of Elf Bar, socio-demographic characteristics and dependence indicators and reasons for use over other brands.

**Design:**

Cross-sectional survey.

**Setting and participants:**

Online 2022 International Tobacco Control Project Youth Tobacco and Vaping Survey (*N* = 1355 16–29-year-olds in England who had vaped in the past 30 days).

**Measurements:**

Currently using Elf Bar most often (versus other brands) and associations with: socio-demographics, owning a vaping device, dependence indicators and reasons for brand choice. Logistic regressions were used.

**Findings:**

Among 16–29-year-olds who vaped in the past 30 days, 48.4% (*n* = 732) reported Elf Bar as the brand they used most often. Among 16–17-year-olds, 40.7% used Elf Bar over other brands; this was lower than among 18–19-year-olds (60.1%) and 20–29-year-olds (47.4%) (*P* ≤ 0.002). Using Elf Bar over other brands was higher among those who were female (55.2 versus 41.5% male), identified as White (53.1 versus 30.9% other/mixed), a student (54.5 versus 44.3% not), did not own a vape (66.7 versus 44.4% who did) and typically vaped 5–8 hours after waking (62.7 versus 36.8% within 5 min) (*P* ≤ 0.044). Most who vaped but had never smoked used Elf Bar (64.3%), although use did not significantly differ from those who currently (45.4%), formerly (42.3%) or experimentally (48.7%) smoked (all *P* ≥ 0.060). Popular reasons for choosing Elf Bar over other brands were better flavour/taste (47.5%), less expensive (28.7%), easier to get (26.1%), smoother to inhale (24.0%) and popularity (23.1%). ‘Better for quitting smoking’ (10.1%) was least frequently selected reason for choosing Elf Bar over other brands.

**Conclusions:**

Elf Bar brand e-cigarettes were used by approximately half of 16–29-year-olds who vaped in England in 2022 and was mainly chosen over other brands for subjective responses (e.g. flavour/taste), rather than for quitting smoking.

## INTRODUCTION

The vaping (e-cigarette) market in Great Britain has rapidly evolved since 2021 with the introduction of novel disposables, such as Elf Bars [1, 2]. The proportion of youth aged 11–17 years who vape disposable e-cigarettes, compared with other vaping devices, was 69% in 2023, an increase from 8% in 2021 [1]. Similar increases were observed among 18-year-olds between 2021 and 2022 [2].

Despite policy measures to reduce youth access to nicotine products (e.g. minimum legal age of sale of 18 years, including proxy purchasing) [3], there are concerns that novel disposable vapes are particularly appealing and accessible to youth [2, 4]. Disposable vapes are available in a range of flavours and colours and are convenient to use because they come fully charged and pre-filled with e-liquid [2, 5]. They are also currently widely available in retailers throughout Great Britain, including convenience shops and supermarkets, and are often advertised at the point of sale [2]. Qualitative research has suggested that 11–16-year-olds in the United Kingdom perceive disposable vapes to be targeted towards them, while other e-cigarette types (e.g. tanks) are perceived to be used more by older adults [5]. In January 2024, UK government announced a ban on disposable vapes, and this will require development of new regulations.

Elf Bar was the highest grossing vaping brand in 2022 according to UK sales data [6]. Elf Bar was also the most popular brand of disposable vape according to survey data, used by approximately half of youth aged 11–17 years who vape [1] and approximately half of adults aged 18 or over who mainly vape disposables [7] in Great Britain in 2023. Elf Bars in the United Kingdom typically contain prefilled e-liquid and are available in a range of flavours and colours, widely available, affordable (typically retailing at ~£5, although lower prices have been recorded) and often available on a multi-buy offer [8]. Elf Bars also typically contain 20 mg/ml of nicotine salt e-liquid (the maximum allowed in the United Kingdom) [9], so there is concern that they could promote and sustain nicotine dependence. In early 2023, some flavoured Elf Bar vapes were recalled from retailers as a result of containing a larger capacity tank and hence more nicotine volume than is legally permitted in the United Kingdom [10, 11]. While larger tanks do not necessarily increase health risks, they may increase duration of use and therefore require less frequent purchasing than other disposable brands, which may be particularly appealing to underage youth.

Most youth aged 11–17 years in Great Britain who have tried vaping report doing so ‘just to give it a try’, followed by ‘other people use them so I join in’, then for liking the flavours [1]. Qualitative research among youth further suggests that ease of purchasing (including low cost), range of flavours and discreet design were commonly endorsed reasons for trying disposable vapes specifically [5]. In England in 2020, adults were more likely to report quitting smoking as a reason for vaping if they used tanks or cartridge/pod devices than disposable devices [12], although this was before novel disposables became prevalent. The vast majority of youth and young adults who vape in Great Britain also currently smoke or used to smoke, although recently there has been an increase in vaping prevalence among people who have never smoked [1, 13]. Indicators of dependence (e.g. urges to vape) have also increased in recent years among 16–19-year-olds who vape [14, 15]. To our knowledge, there have been no studies assessing reasons for Elf Bar use specifically—given the increases in vaping among people who have never smoked and popularity of Elf Bar, it is important to address this gap.

This paper therefore aims to examine, among youth and young adults in England who had vaped in the past 30 days, (1) the prevalence of Elf Bar use, (2) socio-demographic characteristics and dependence indicators among those who vaped Elf Bar compared with other brands of vaping products and (3) reasons for brand choice among those who vaped Elf Bar compared with other brands of vaping products.

## METHODS

### Data source

**FIGURE 1:**
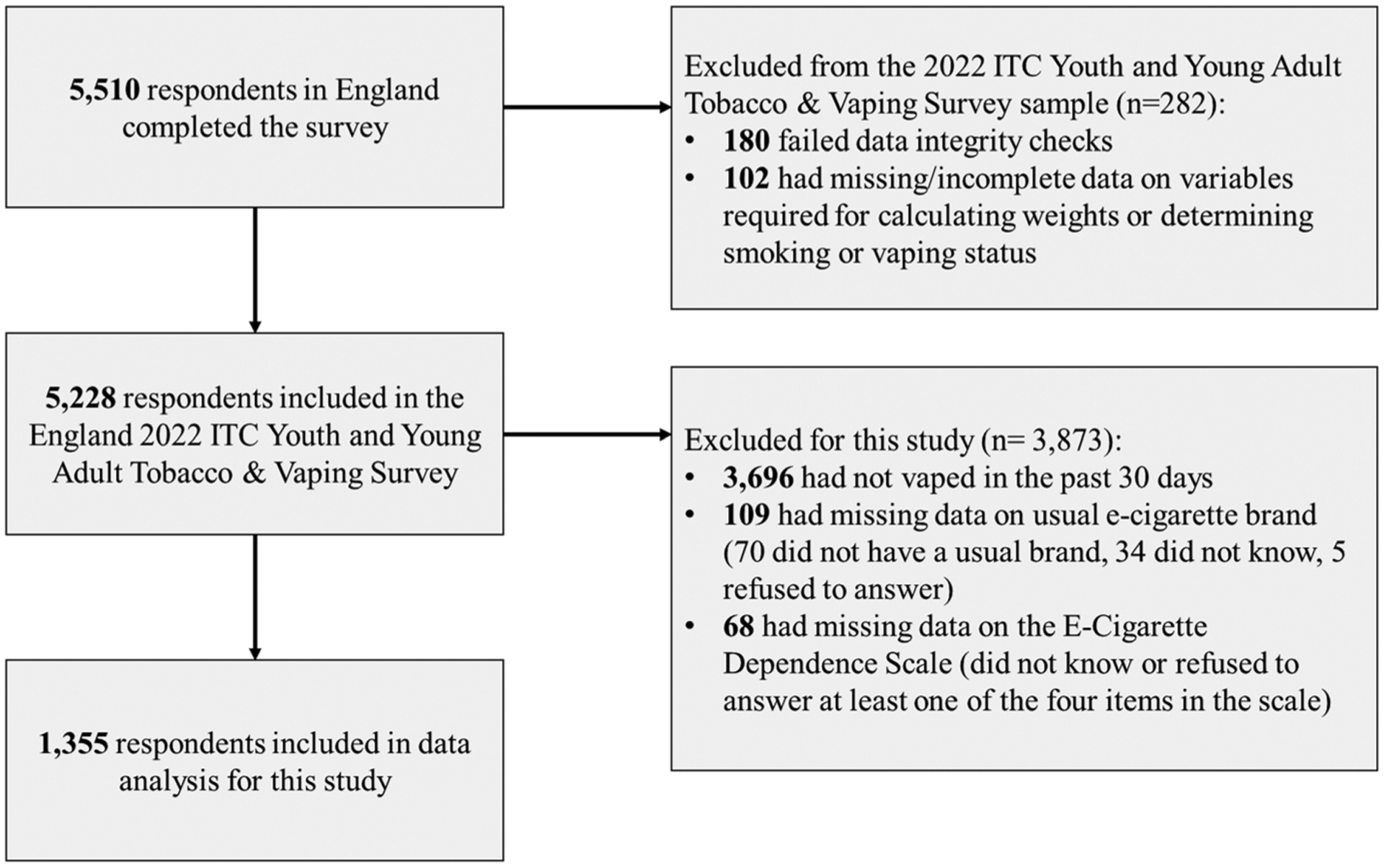
Strengthening the Reporting of Observational studies in Epidemiology (STROBE) flow-chart of sample recruitment and exclusion.

Data were from the England 2022 arm of the International Tobacco Control (ITC) Policy Evaluation Project Youth Tobacco and Vaping Survey, a cross-sectional on-line survey of youth and young adults aged 16–29 years (with sampling focused upon 16–19-year-olds in earlier survey waves). Data were collected between 2 August and 12 September 2022. A full description of the study methods can be found in the Technical Report [16]. A total of 5510 respondents in England completed the survey, of whom 1355 who had vaped in the past 30 days reported having a usual brand of vape, and did not have missing data on the E-cigarette dependence scale (EDS) were retained in the analytical sample for this study (Figure 1). Respondents were required to report their usual brand of vape to derive the outcome ‘currently use Elf Bar most often’ and because respondents who did not report a usual brand of vape were not asked their reasons for brand choice.

### Measures

#### Currently use Elf Bar most often (versus other vape brands)

Respondents who had vaped in the past 30 days were asked: ‘What specific brand of e-cigarette/vaping device do you currently use most often?’, with a country-specific list of 20 brands displayed, including ‘Elf Bar’ [16]. Respondents could also select ‘Other brand (please specify)’ with a free-text option to specify. Respondents who selected ‘Elf Bar’ or ‘Other brand (please specify)’ and entered a variation of Elf Bar [‘Elf’ (*n* = 1), ‘Elf Bar and Voopoo Drag 3’ (*n* = 1), ‘elfbar’ (*n* = 1) or ‘Lost Mary’ (*n* = 3)] into the free-text box were coded as currently using Elf Bar most often. All other respondents were coded as ‘Other’. Supporting information, Table S1 shows the full list of brands and their frequencies.

#### Independent variables: socio-demographic characteristics

##### Age group.

The sample group was aged 16–17, 18–19 and 20–29 years; consistent with prior work [17, 18], age of sale of vaping products and because the 20–to 29-year-old sample was small.

##### Sex. male, female. 

Sex was coded from sex at birth for most respondents, or imputed from gender where sex at birth was missing (see Technical Report [16]).

##### Race/ethnicity.

White only, any other/mixed race/ethnicity (grouped due to low numbers), do not know/refused. Race/ethnicity-was derived from country-specific items (see Technical Report [16]).

##### Current or returning student.

Yes (enrolled currently or for upcoming year), no, do not know/refused.

##### Perceived family financial situation.

Respondents were asked ‘How would you describe your family’s financial situation?’ with response options ‘not meeting basic expenses’, ‘just meeting basic expenses’, ‘meeting needs with a little left over’, ‘living comfortably’ and do not know/refused (‘do not know’, ‘refused’; combined for analyses).

##### Smoking status.

Current (smoked in the past 30 days and smoked 100+ cigarettes in life-time), former (not smoked in the past 30 days and smoked 100+ cigarettes in life-time), experimental (smoked in the past 30 days and smoked < 100 cigarettes in life-time), never (never smoked a cigarette), unclassified.

##### Own a vaping device.

Respondents were asked ‘Do you own an e-cigarette/vaping device?’ with response options ‘yes’, ‘no’ and don’t know/refused (‘don’t know’, ‘refused’; combined for analyses).

##### Independent variables.

Vaping dependence indicators.

##### Last time vaped.

Respondents were asked: ‘When was the last time you used an e-cigarette/vaped?’ with valid response options for the analyses in this paper being ‘earlier today’, ‘not today but sometime in the past 7 days’ and ‘not in the past 7 days but sometime in the past 30 days’. Respondents could select other response options (e.g. ‘5 or more years ago’), but in so doing were excluded from the current analyses.

##### Urges to vape.

Respondents were asked: ‘In the past 30 days, how often did you have a strong urge to use an e-cigarette/vape?’, with response options ‘several times a day’, ‘every day or most days’, ‘at least once a week’, ‘less than once a week’, ‘never’ and ‘do not know/refused’.

##### Time to first vape.

Respondents were asked ‘How soon after waking do you first use an e-cigarette/vape?’, with response options ‘within 5 minutes’, ‘6–30 minutes’, ‘31–60 minutes’, ‘1–4 hours (i.e. in the morning)’, ‘5–8 hours (i.e. in the afternoon)’, ‘more than 8 hours’ and ‘do not know/refused’.

##### EDS.

The four-item EDS is a validated measure of vaping dependence [19]. Respondents were asked: ‘For each of the following statements, please choose the option that best describes you’ followed by four items: (1) ‘I find myself reaching for my e-cigarette without thinking about it’; (2) ‘I drop everything to go out and get e-cigarettes or e-juice’; (3) ‘I vape more before going into a situation where vaping is not allowed’; and (4) ‘when I haven’t been able to vape for a few hours, the craving gets intolerable’. Response options for each were ‘never’ [1]; ‘rarely’ [2]; ‘sometimes’ [3]; ‘often’ [4]; ‘almost always’ [5]; ‘do not know’; ‘refused’. ‘Do not know’ and ‘refused’ responses were coded as missing and, as described above, excluded from all analyses. The ratings from each item were summed to calculate the total score for each respondent (minimum 4, maximum 20), with higher scores indicating greater dependence. The EDS had good reliability among our sample (Cronbach’s alpha 0.833).

##### Reasons for brand choice.

Respondents were asked: ‘What are the main reasons you chose to use this brand of e-cigarettes instead of other brands?’ followed by a list shown in random order (select all that apply): ‘better for quitting smoking’, ‘better-looking’, ‘easier to use’, ‘easier to hide’, ‘easier to get’, ‘better flavour/taste’, ‘more fun’, ‘stronger nicotine “hit”‘, ‘less expensive’, ‘more popular among friends’, ‘less harmful’, ‘I was offered it’, ‘smoother to inhale’, do not know/refused (‘do not know’, ‘refused’; combined for analyses). Respondents could also select ‘Other reason(s) (please specify)’, and responses were recoded into the aforementioned list where appropriate.

##### Currently use disposable vaping devices most often (sensitivity analyses).

To assess whether the findings for the brand-derived measure of Elf Bar use applied to disposables generally, a sensitivity analysis was conducted using a second outcome measure. Respondents who had vaped in the past 30 days were asked ‘Which of the following TYPES of e-cigarettes/vaping devices do you currently use *most often*?’ with response options ‘Disposable (not refillable or rechargeable) e-cigarette/vaping device’, ‘E-cigarette/vaping device with replaceable prefilled cartridges or pods’, ‘E-cigarette/vaping device with a tank that you fill with liquid’, ‘don’t know’ and ‘refused’. For the purposes of sensitivity analyses, responses were coded as disposable versus otherwise.

### Analyses

First, to examine prevalence of Elf Bar use, the number and proportion of youth and young adults who had vaped in the past 30 days and who reported currently using Elf Bar most often (compared with other brands) was reported (aim 1). Secondly, to examine socio-demographics and dependence indicators of youth and young adults who usually vape Elf Bar, unadjusted and adjusted logistic regression models were used to predict currently using Elf Bar most often (aim 2); models are detailed in the footnote of Table 1—briefly, one model was run mutually adjusting for socio-demographics, smoking status and owning a vaping device (to adjust for confounding); then, due to concerns over collinearity of the four vaping dependence indicators and to avoid over-adjusting for vaping dependence, four individual models were run with each of the four dependence indicators separately while adjusting for socio-demographics, smoking status and owning a vaping device. Thirdly, to examine reasons for brand choice, the number and proportion who reported each reason for use were reported among youth and young adults who currently used Elf Bar most often compared with other vape brands, and proportions were compared using unadjusted and adjusted (for socio-demographics and smoking status) logistic regression models (aim 3). Sensitivity analyses repeated all steps with the outcome of currently using disposable devices most often. Analyses were conducted in Stata version 17 and applied cross-sectional post-stratification sample weights to all data. The 16–19-year-olds were weighted separately from the 20–29-year-olds, and so a rescaled weight that combined inflation weights from both age groups was used (see Technical Report) [16]. Analyses were not pre-registered so results are exploratory.

## RESULTS

### Sample characteristics

Among youth and young adults who had vaped in the past 30 days, most (*n* = 774) were aged 18–19 years, while *n* = 303 were aged 16–17 and *n* = 278 were aged 20–29. When sample weights were applied to enhance the representativeness of the sample, respondents aged 20–29 years represented most of the sample (79.9%), while ages 16–17 and 18–19 represented 8.0 and 12.1%, respectively. Approximately half were female (50.2%), and the majority were classified as white (only) race/ethnicity (78.7%), were not students (61.0%) and perceived their family’s financial situation as either meeting needs with a little left over (35.1%) or just meeting basic expenses (34.4%). Approximately half were classified as an ‘experimental smoker’ (49.7%), one-quarter currently smoked (29.2%) and 10.3% reported never having tried smoking. The majority also reported vaping within the last 7 days (70.8%, of whom 39.9% had vaped earlier today) and had strong urges to vape at least once a week (76.2%), and the mean EDS score was 10.4 [95% confidence interval (CI) = 9.9–10.8], indicating moderate dependence [19].

### Prevalence of Elf Bar use (aim 1)

Among youth and young adults who had vaped in the past 30 days, approximately half reported Elf Bar as the brand they currently used most often (48.4%, *n* = 732).

### Socio-demographic characteristics and dependence indicators of youth and young adults who vaped Elf Bar compared with other brands (aim 2)

In unadjusted and adjusted analyses, youth and young adults who had vaped in the past 30 days had higher odds of reporting Elf Bar as the brand they currently used most often, compared with other brands, if they were older, female, identified as white (only) race/ethnicity, and did not own a vaping device (Table 1). Changing the reference categories, odds of using Elf Bar were also higher among those age 18–19 years in unadjusted analyses only [versus 20–29: odds ratio (OR) = 1.68, 1.20–2.33, *P* = 0.002] (data not shown in tables). Being a current or returning student was associated with higher odds of using Elf Bar in adjusted analyses only (Table 1).

Most of the sample who had vaped in the past 30 days but had never smoked reported Elf Bar as the brand they currently used most often (64.3%), although use did not differ statistically significantly from those who currently (45.4%) or formerly (42.3%) smoked or had experimented (48.7%) with smoking (all *P* ≥ 0.060) (Table 1).

Considering dependence indicators, the odds of reporting Elf Bar as the brand currently used most often were higher among those who reported longer time to first use after waking (5–8 hours versus within 5 minutes), but there were no significant differences between other dependence indicators and Elf Bar use (Table 1). Changing the reference categories, odds of using Elf Bar were also higher among those who reported vaping 5–8 hours versus 1–4 hours after waking [unadjusted: OR = 2.64, 1.22–5.71, *P* = 0.014; adjusted odds ratio (AOR) = 3.10, 1.40–6.87, *P* = 0.005) (data not shown in tables).

### Reasons for brand choice (aim 3)

The most popular reasons for brand choice among those who used Elf Bar were ‘better flavour/taste’ (47.5%), ‘less expensive’ (28.7%), ‘easier to get’ (26.1%), ‘smoother to inhale’ (24.0%) and ‘more popular among friends’ (23.1%). ‘Better for quitting smoking’ (10.1%) was the least frequently selected reason.

‘Better flavour/taste’ was selected more frequently among youth and young adults who used Elf Bar over other brands in both unadjusted and adjusted analyses, while ‘less expensive’ was only more frequently selected in unadjusted analyses (Table 2). Other reasons for brand choice did not differ significantly between those who used Elf Bar compared with other brands (Table 2).

#### Sensitivity analyses

When using the outcome of generally using disposable vaping devices, youth and young adults who had vaped in the past 30 days had higher odds of using disposables if they were aged 18–19 (versus 16–17) and, in adjusted analyses only, had never smoked (versus formerly smoked; Supporting information, Table S2). Changing the reference categories, the odds were also higher among those classified as ‘experimental smokers’ (versus formerly smoked, unadjusted: OR = 2.62, 1.14–6.01, *P* = 0.024; adjusted AOR = 2.89, 1.10–7.63, *P* = 0.032) (data not shown in tables).

Consistent with the primary analyses using Elf Bar as an outcome, ‘better flavour/taste’ (41.1%), ‘less expensive’ (24.5%), ‘easier to get’ (23.9%) and ‘smoother to inhale’ (21.8%) were the most frequently endorsed reasons for brand choice among those who used disposables generally, while ‘better for quitting smoking’ (12.7%) was the least frequently endorsed (Supporting information, Table S3). ‘Better flavour/taste’ was also more frequently selected among those who used disposable vapes than other device types (Supporting information, Table S3).

## DISCUSSION

This is the first research study, to our knowledge, to specifically examine use of Elf Bar vapes among youth and young adults. Consistent with national surveys [1, 2], approximately half of youth and young adults who vaped used Elf Bar more often than other vaping devices in England in 2022. Previously published data from the same study found that no 16–19-year-olds in England reported Elf Bar use in 2017 to 2020 [20] and, in 2021, only 1% of 16–19-year-olds who vaped reported using Elf Bar most often [21]; findings therefore highlight the rapid rise of Elf Bar in a 12-month period.

Consistent with concerns expressed in the literature [2] and by the UK government [4], the use of Elf Bar, and disposables in general, was common among youth and young adults who vaped but had never smoked, and approximately 40% of youth aged 16–17 years who vape used Elf Bar most often. However, Elf Bar use among those who had never smoked was not significantly different from among those who smoke, and compared to 16–17-year-olds use was more common among those who were of legal purchasing age (18+ years). ‘Better for quitting smoking’ was the least frequently endorsed reason for choosing Elf Bar, and although this was not significantly different from reasons for choosing other brands, was similar among those who used disposable products in general and may be explained by fewer current/former smokers aged 16–29 using Elf Bar.

Also consistent with prior research [5] and concerns in the literature [2] and of UK government [4], better flavour/taste, low cost and ease of access were common reasons for choosing Elf Bar as well as disposables in general. These findings may reflect disposables retailing at low prices and often in multi-buy offers.

However, Elf Bar may have some of its own unique customer demographics and appeal. Elf Bars (but not disposables in general) were more frequently used by youth and young adults who identified as female, White and a student. Elf Bars are popular for their flavour/taste, and young adults who believe that vaping certain flavours can help with appetite control are more likely to initiate vaping [22], which may partially explain the sex differences observed in our study. Those who used Elf Bar, compared with other brands, also less frequently self-reported owning an e-cigarette/vaping device; this finding may suggest that Elf Bars are more likely to be shared among peer groups, that use is more experimental than for other brands and that youth and young adults do not consider themselves to ‘own’ an Elf Bar.

There was little clear evidence that nicotine dependence was higher among youth and young adults who primarily used Elf Bar compared with other brands of vapes; rather, using Elf Bar over other brands was associated with vaping in the afternoon rather than upon waking. Moreover, ‘stronger nicotine hit’ was endorsed as a reason for use by only 12% of those who used Elf Bar most often, a proportion similar among users of other brands (12%) and those who used disposables in general (11%). These findings may reflect more experimental use of Elf Bar (e.g. less likely to own a vaping device, potential sharing among peer groups in the afternoons) and/or that Elf Bar and novel disposables are relatively new to the market and so transitions to more frequent use may not have had time to occur; indeed, research suggests that nicotine intake from vapes is lower among those with less vaping experience [23].

Limitations of this study include its cross-sectional nature, which precludes determining the direction of associations (e.g. between dependence indicators and Elf Bar use), self-reported items, which may be subject to misreporting and samples that were not probability-based and crude categorization of some covariates (e.g. race/ethnicity). Data were also among youth and young adults in England, so findings may not generalize to older adults or other countries. Strengths include the use of data from underage youth as well as those of legal purchasing age (18+), sensitivity analyses to examine whether findings applied to disposables in general and a large sample of youth and young adults who had vaped in the past 30 days to examine use of Elf Bar in depth.

To conclude, Elf Bar has altered the vaping product market in England, with high levels of use among youth and young adults who vape. Some of the popularity may be attributable to Elf Bars’ unique attributes; however, a combination of factors that are increasingly observed in vaping products more generally, including low cost, wide-spread retail availability, attractive imagery, appealing flavours and a shift towards salt-based e-liquid that facilitates nicotine inhalation, is probably contributing to increases in appeal [2, 5, 17]. Recently, Elf Bar has launched rechargeable/refillable models with many of the same attributes as their disposable models. Given the rapidly evolving market, policy measures to reduce the appeal and access of vapes to underage and non-smoking youth should address youth-appealing product attributes in general, rather than addressing a single product (e.g. Elf Bar); these could include better enforcement of age-of-sale laws, restricting brand imagery and advertisements at the point of sale and reducing affordability (e.g. increasing taxation), although measures should not restrict access or appeal to a greater extent than tobacco cigarettes.

## Supplementary Material

Supplementary material

## Figures and Tables

**TABLE 1:** Sample characteristics and logistic regression analyses predicting currently using Elf Bar most often (versus other brands) among youth and young adults who have vaped in the past 30 days in England, 2022. All data except *n* are weighted.

	*N* (%)			Use Elf Bar (versus other brands)			
	Full sample, vaped in past 30 days (*n* = 1355)	Use other brand(s) most often (*n* = 623, 51.63%)	Use Elf Bar most often (*n* = 732, 48.37%)	Unadjusted		Adjusted	
				OR (95% CI)	*P*	AOR (95% CI)	*P*
Age group (years)^a^							
16–17	303 (7.97)	179 (59.26)	124 (40.74)	1.00		1.00	
18–19	774 (12.10)	303 (39.87)	471 (60.13)	**2.19 (1.63–2.95)**	**< 0.001**	**2.56 (1.76–3.74)**	**< 0.001**
20–29	278 (79.92)	141 (52.65)	137 (47.35)	1.31 (0.90–1.91)	0.163	**2.64 (1.44–4.83)**	**0.002**
Sex^a^							
Male	377 (49.76)	206 (58.54)	171 (41.46)	1.00		1.00	
Female	978 (50.24)	417 (44.8)	561 (55.20)	**1.74 (1.09–2.78)**	**0.021**	**1.91 (1.20–3.05)**	**0.007**
Race/ethnicity^a^							
Any other/mixed	226 (20.03)	114 (69.10)	112 (30.90)	1.00		1.00	
White only	1121 (78.74)	505 (46.93)	616 (53.07)	**2.53 (1.41–4.55)**	**0.002**	**2.77 (1.52–5.08)**	**0.001**
Do not know/refused	8 (1.232)	4 (68.45)	4 (31.55)	1.03 (0.13–7.91)	0.976	0.39 (0.07–2.06)	0.266
Current or returning student^a^							
No	471 (61.01)	226 (55.67)	245 (44.33)	1.00		1.00	
Yes	875 (38.79)	394 (45.5)	481 (54.50)	1.50 (0.96–2.36)	0.077	**1.82 (1.02–3.25)**	**0.044**
Do not know/refused	9 (0.20)	3 (11.27)	6 (88.73)	**9.89 (1.32–74.04)**	**0.026**	**8.85 (1.22–64.08)**	**0.031**
Perceived family financial situation^a^							
Living comfortably	334 (17.88)	182 (55.68)	152 (44.32)	1.00		1.00	
Not meeting basic expenses	114 (10.04)	50 (50.57)	64 (49.43)	1.23 (0.53–2.86)	0.634	1.30 (0.54–3.13)	0.553
Just meeting basic expenses	450 (34.42)	192 (57.31)	258 (42.69)	0.94 (0.51–1.72)	0.830	0.93 (0.51–1.68)	0.800
Meeting needs with a little left over	417 (35.07)	184 (46.90)	233 (53.10)	1.42 (0.77–2.64)	0.263	1.43 (0.76–2.69)	0.264
Do not know/refused	40 (2.59)	15 (16.54)	25 (83.46)	**6.34 (1.79–22.5)**	**0.004**	**6.26 (1.25–31.34)**	**0.026**
Smoking status^a^							
Never	182 (10.26)	74 (35.66)	108 (64.34)	1.00		1.00	
Current	361 (29.22)	195 (54.65)	166 (45.35)	0.46 (0.20–1.03)	0.060	0.48 (0.18–1.33)	0.161
Former	77 (9.96)	37 (57.73)	40 (42.27)	0.41 (0.15–1.11)	0.078	0.33 (0.11–1.05)	0.061
Experimental	724 (49.71)	312 (51.33)	412 (48.67)	0.53 (0.24–1.14)	0.102	0.56 (0.21–1.50)	0.252
Do not know/refused	11 (0.85)	5 (87.33)	6 (12.67)	0.08 (0.01–0.47)	0.005	0.19 (0.03–1.13)	0.068
Own a vaping device^a^							
No	336 (22.04)	127 (33.29)	209 (66.71)	1.00		1.00	
Yes	985 (74.05)	474 (55.57)	511 (44.43)	**0.40 (0.23–0.70)**	**0.001**	**0.46 (0.25–0.83)**	**0.010**
Do not know/refused	34 (3.91)	22 (80.59)	12 (19.41)	**0.12 (0.03–0.42)**	**0.001**	**0.15 (0.03–0.63)**	**0.010**
Last time vaped^b^							
Not in last 7 days but sometime in last 30 days	354 (29.17)	174 (55.49)	180 (44.51)	1.00		1.00	
Not today but sometime in last 7 days	402 (30.97)	178 (52.35)	224 (47.65)	1.34 (0.76–2.34)	0.309	1.32 (0.70–2.46)	0.389
Earlier today	599 (39.85)	271 (48.26)	328 (51.74)	1.13 (0.61–2.12)	0.692	1.65 (0.88–3.08)	0.117
Urges to vape^c^							
Never	226 (13.80)	82 (44.21)	144 (55.79)	1.00		1.00	
Less than once a week	152 (8.34)	55 (42.29)	97 (57.71)	1.08 (0.42–2.80)	0.872	1.40 (0.54–3.63)	0.485
At least once a week	283 (22.52)	151 (57.66)	132 (42.34)	0.58 (0.26–1.30)	0.187	1.04 (0.45–2.41)	0.933
Every day or most days	317 (26.39)	161 (53.68)	156 (46.32)	0.68 (0.31–1.51)	0.347	0.97 (0.42–2.20)	0.932
Several times a day	364 (27.30)	169 (52.49)	195 (47.51)	0.72 (0.32–1.60)	0.416	1.03 (0.44–2.39)	0.948
Do not know/refused	13 (1.66)	5 (31.93)	8 (68.07)	1.69 (0.2–14.42)	0.631	4.44 (0.73–26.94)	0.105
Time to first vape^d^							
Within 5 minutes	229 (14.12)	119 (63.19)	110 (36.81)	1.00		1.00	
6–30 minutes	269 (20.6)	122 (54.79)	147 (45.21)	1.42 (0.64–3.15)	0.393	1.37 (0.63–2.98)	0.421
31–60 minutes	203 (19.37)	108 (52.37)	95 (47.63)	1.56 (0.68–3.60)	0.295	1.42 (0.62–3.24)	0.403
1–4 hours (i.e. in the morning)	244 (17.8)	126 (61.08)	118 (38.92)	1.09 (0.49–2.45)	0.827	0.86 (0.40–1.86)	0.697
5–8 h (i.e. in the afternoon)	218 (16.41)	81 (37.32)	137 (62.68)	**2.88 (1.24–6.73)**	**0.014**	**2.66 (1.15–6.16)**	**0.022**
More than 8 hours	118 (6.76)	41 (38.98)	77 (61.02)	2.69 (0.96–7.55)	0.061	1.31 (0.47–3.64)	0.603
Do not know/refused	74 (4.94)	26 (33.35)	48 (66.65)	**3.43 (1.08–10.95)**	**0.037**	3.17 (0.92–10.94)	0.068
E-cigarette dependence scale (mean 95% CI)^e^	10.4 (9.9–10.8)	10.5 (10.0–11.1)	10.2 (9.5–10.8)	0.98 (0.92–1.03)	0.391	1.01 (0.95–1.07)	0.761

All data are weighted, except *n* which are unweighted. Bolded associations are those that are statistically significant at *p* < 0.05.

a Adjusted logistic regression models include age group, sex, race/ethnicity, current or returning student, perceived financial status, smoking status and own a vaping device.

b Adjusted logistic regression model includes last time vaped, age group, sex, race/ethnicity, current or returning student, perceived financial status, smoking status and own a vaping device.

c Adjusted logistic regression model includes urges to vape, age group, sex, race/ethnicity, current or returning student, perceived financial status, smoking status and own a vaping device.

d Adjusted logistic regression model includes time to first vape, age group, sex, race/ethnicity, current or returning student, perceived financial status, smoking status and own a vaping device.

e Adjusted logistic regression model includes E-cigarette dependence scale, age group, sex, race/ethnicity, current or returning student, perceived financial status, smoking status and own a vaping device. AOR = adjusted odds ratio; CI = confidence interval.

**TABLE 2:** Reasons for brand choice among youth and young adults who have vaped in the past 30 days among those who used Elf Bar versus otherwise in England, 2022. Reasons are not mutually exclusive. All data except *n* are weighted (*n* = 1355).

	*n* (%)			Use Elf Bar (versus other brands)			
	Full sample, vaped in past 30 days (*n* = 1355)	Use other brand(s) most often (*n* = 623, 51.63%)	Use Elf Bar most often (*n* = 732, 48.37%)	Unadjusted		Adjusted	
				OR (95% CI)	*P*	AOR (95% CI)^a^	*P*
Better flavour/taste	576 (36.47)	217 (26.13)	359 (47.51)	**2.56 (1.59–4.11)**	**< 0.001**	**2.64 (1.64–4.24)**	**< 0.001**
Less expensive	444 (23.77)	170 (19.11)	274 (28.74)	**1.71 (1.09–2.68)**	**0.021**	1.49 (0.94–2.37)	0.090
Easier to get	387 (23.82)	157 (21.69)	230 (26.10)	1.28 (0.78–2.09)	0.337	1.20 (0.74–1.94)	0.465
Smoother to inhale	334 (21.03)	159 (18.3)	175 (23.95)	1.41 (0.80–2.46)	0.234	1.35 (0.77–2.38)	0.291
More popular among friends	362 (19.3)	122 (15.71)	240 (23.14)	1.62 (0.94–2.77)	0.081	1.66 (0.95–2.90)	0.074
Easier to use	327 (24.03)	163 (26.72)	164 (21.15)	0.74 (0.44–1.23)	0.245	0.69 (0.41–1.17)	0.168
Better-looking	230 (19.63)	114 (20.17)	116 (19.04)	0.93 (0.51–1.68)	0.813	1.08 (0.61–1.91)	0.787
I was offered it	195 (14.08)	100 (15.16)	95 (12.93)	0.83 (0.43–1.62)	0.587	0.73 (0.37–1.44)	0.369
Stronger nicotine hit	175 (12.07)	95 (11.87)	80 (12.28)	1.04 (0.53–2.05)	0.913	1.06 (0.50–2.26)	0.882
Easier to hide	153 (11.47)	74 (10.78)	79 (12.21)	1.15 (0.58–2.30)	0.688	1.09 (0.52–2.30)	0.815
More fun	160 (14.93)	84 (13.53)	76 (16.43)	1.26 (0.65–2.41)	0.493	1.45 (0.72–2.91)	0.298
Less harmful	139 (13.49)	77 (15.1)	62 (11.77)	0.75 (0.36–1.57)	0.445	0.66 (0.31–1.42)	0.288
Better for quitting smoking	158 (13.3)	96 (16.28)	62 (10.13)	0.58 (0.31–1.10)	0.094	0.51 (0.26–1.03)	0.059
Do not know/refused	27 (0.94)	11 (0.84)	16 (1.04)	1.25 (0.28–5.58)	0.770	1.30 (0.44–3.81)	0.635

All data are weighted, except *n* that are unweighted. Bolded associations are those that are statistically significant at *p* < 0.05.

a Adjusted logistic regression models include, age group, sex, race/ethnicity, current or returning student, perceived financial status and smoking status. CI = confidence interval; OR = odds ratio.

## Data Availability

Enquiries regarding data availability should be emailed to the PI, Professor David Hammond, at david.hammond@uwaterloo.ca.
