# Supplementary material for: Use of ‘Elf Bar’ among youth and young adults who currently vape in England: cross‐sectional associations with demographics, dependence indicators and reasons for use
Source: Addiction. 2024 Mar 21;120(3):414–22. doi: 10.1111/add.16463 (PMC11415542; doi:10.1111/add.16463)
Supplement: Supplementary file 1 — Table A1 Brand of e‐cigarette/vaping device currently used most often among youth and young adults who vaped in the past 30 days in England, 2022. N = 1,355. [file ADD-120-414-s001.docx]

**Table A1. Brand of e-cigarette/vaping device currently used most often among youth and young adults who vaped in the past 30 days in England, 2022. N = 1,355.**

|  | Unweighted n | Weighted % |
| --- | --- | --- |
| Elf Bar | 732 | 48.37 |
| Geek Bar | 90 | 6.48 |
| Elux* | 86 | 3.62 |
| Smok (including Nord, Novo) | 57 | 4.61 |
| JUUL | 50 | 1.69 |
| Blu | 47 | 6.57 |
| Aspire | 44 | 4.34 |
| 88 Vape | 41 | 3.81 |
| Vuse/Vype | 26 | 2.85 |
| Solo | 25 | 1.21 |
| Geek Vape | 22 | 2.50 |
| Voopoo (including Drag) | 22 | 1.25 |
| Puff Bar | 19 | 1.32 |
| eGo | 12 | 1.45 |
| Other brand | 12 | 0.69 |
| Eleaf | 11 | 2.69 |
| E-Lites | 10 | 2.35 |
| VIP | 7 | 0.78 |
| Innokin* | 6 | 0.11 |
| RandM* | 6 | 0.69 |
| Logic | 5 | 0.63 |
| UWELL Caliburn* | 4 | 0.06 |
| Vaporesso* | 4 | 0.31 |
| Totally Wicked* | 3 | 0.06 |
| Aroma King* | 2 | 0.05 |
| Dot* | 2 | 0.07 |
| Hyde | 2 | 0.03 |
| Lost Vape* | 2 | 0.01 |
| OXVA* | 2 | 0.03 |
| Dinner Lady* | 1 | 0.61 |
| Freemax* | 1 | 0.25 |
| Kanger Tech | 1 | 0.46 |
| Vivid* | 1 | 0.04 |

*Item created based on written response. All other items were displayed on a drop-down list from which respondents could select the brand they used most often.

| **Table A2. Logistic regression analyses predicting currently using disposable vapes most often (vs. otherwise) among youth and young adults who have vaped in the past 30 days in England, 2022. All data except N are weighted. N = 1,355.** | | | | | | | | | | | | | | | | | |  |  |  |  |
| --- | --- | --- | --- | --- | --- | --- | --- | --- | --- | --- | --- | --- | --- | --- | --- | --- | --- | --- | --- | --- | --- |
|  |  | **N (%)** | | | | |  | **Use disposables (vs. otherwise)** | | | | | | | | | |  |  |  |  |
|  |  | **Responded otherwise**  **(N=434, 33.75%)** | | **Use disposables most often**  **(N=921, 66.25%)** | | |  | **Unadjusted** | | | |  | **Adjusted** | | | | |  |  |  |  |
|  |  |  |  |  |  |  |  | **OR (95% CI)** | | | **p** |  | **AOR (95% CI)** | | | **p** | |  |  |  |  |
| **Age Group^1^** |  |  | |  | | |  |  | | |  |  |  | | |  | |  |  |  |  |
| 16-17 |  | 120 (39.97) | | 183 (60.03) | | |  | 1.00 | | |  |  | 1.00 | | |  | |  |  |  |  |
| 18-19 |  | 211 (27.18) | | 563 (72.82) | | |  | **1.78 (1.31-2.43)** | | | **<.001** |  | **1.79 (1.25-2.54)** | | | **.001** | |  |  |  |  |
| 20-29 |  | 103 (34.12) | | 175 (65.88) | | |  | 1.29 (0.87-1.89) | | | .204 |  | 1.66 (0.92-3.00) | | | .091 | |  |  |  |  |
|  |  |  | |  | | |  |  | | |  |  |  | | |  | |  |  |  |  |
| **Sex^1^** |  |  | |  | | |  |  | | |  |  |  | | |  | |  |  |  |  |
| Male |  | 139 (32.29) | | 238 (67.71) | | |  | 1.00 | | |  |  | 1.00 | | |  | |  |  |  |  |
| Female |  | 295 (35.19) | | 683 (64.81) | | |  | 0.88 (0.54-1.43) | | | .604 |  | 0.96 (0.57-1.62) | | | .887 | |  |  |  |  |
|  |  |  | |  | | |  |  | | |  |  |  | | |  | |  |  |  |  |
| **Race/ethnicity^1^** |  |  | |  | | |  |  | | |  |  |  | | |  | |  |  |  |  |
| Any other/mixed |  | 360 (32.05) | | 761 (67.95) | | |  | 1.00 | | |  |  | 1.00 | | |  | |  |  |  |  |
| White only |  | 71 (37.37) | | 155 (62.63) | | |  | 1.27 (0.69-2.32) | | | .448 |  | 1.53 (0.82-2.87) | | | .183 | |  |  |  |  |
| Don't know/refused |  | 3 (83.65) | | 5 (16.35) | | |  | **0.12 (0.01-0.91)** | | | **.041** |  | 0.12 (0.01-1.53) | | | .102 | |  |  |  |  |
|  |  |  | |  | | |  |  | | |  |  |  | | |  | |  |  |  |  |
| **Current or returning student^1^** |  |  | |  | | |  |  | | |  |  |  | | |  | |  |  |  |  |
| No |  | 176 (35.14) | | 295 (64.86) | | |  | 1.00 | | |  |  | 1.00 | | |  | |  |  |  |  |
| Yes |  | 255 (31.71) | | 620 (68.29) | | |  | 1.17 (0.73-1.87) | | | .521 |  | 1.10 (0.60-2.05) | | | .752 | |  |  |  |  |
| Don’t know/Refused |  | 3 (7.02) | | 6 (92.98) | | |  | **7.18 (1.11-46.46)** | | | **.039** |  | **10.2 (1.09-95.07)** | | | **.041** | |  |  |  |  |
|  |  |  | |  | | |  |  | | |  |  |  | | |  | |  |  |  |  |
| **Perceived family financial situation^1^** |  |  | |  | | |  |  | | |  |  |  | | |  | |  |  |  |  |
| Living comfortably |  | 92 (36.20) | | 242 (63.80) | | |  | 1.00 | | |  |  | 1.00 | | |  | |  |  |  |  |
| Not meeting basic expenses |  | 43 (37.65) | | 71 (62.35) | | |  | 0.94 (0.40-2.23) | | | .888 |  | 0.95 (0.38-2.37) | | | .910 | |  |  |  |  |
| Just meeting basic expenses |  | 144 (34.44) | | 306 (65.56) | | |  | 1.08 (0.57-2.06) | | | .815 |  | 1.05 (0.55-2.01) | | | .883 | |  |  |  |  |
| Meeting needs with a little left over |  | 142 (28.99) | | 275 (71.01) | | |  | 1.39 (0.72-2.68) | | | .326 |  | 1.26 (0.64-2.48) | | | .502 | |  |  |  |  |
| Don’t know/Refused |  | 13 (57.03) | | 27 (42.97) | | |  | 0.43 (0.10-1.82) | | | .249 |  | 0.39 (0.05-2.90) | | | .358 | |  |  |  |  |
|  |  |  | |  | | |  |  | | |  |  |  | | |  | |  |  |  |  |
| **Smoking status^1^** |  |  | |  | | |  |  | | |  |  |  | | |  | |  |  |  |  |
| Never |  | 52 (30.59) | | 130 (69.41) | | |  | 1.00 | | |  |  | 1.00 | | |  | |  |  |  |  |
| Current |  | 124 (38.05) | | 237 (61.95) | | |  | 0.72 (0.30-1.71) | | | .454 |  | 0.55 (0.25-1.23) | | | .145 | |  |  |  |  |
| Former |  | 44 (51.01) | | 33 (48.99) | | |  | 0.42 (0.15-1.23) | | | .115 |  | **0.30 (0.10-0.94)** | | | **.040** | |  |  |  |  |
| Experimental |  | 211 (28.48) | | 513 (71.52) | | |  | 1.11 (0.47-2.58) | | | .814 |  | 0.87 (0.41-1.85) | | | .720 | |  |  |  |  |
| Don't know/refused |  | 3 (30.11) | | 8 (69.89) | | |  | 1.02 (0.09-11.65) | | | .985 |  | 1.19 (0.11-12.39) | | | .883 | |  |  |  |  |
| *Table A2 continued below.* |  |  | |  | | |  |  | | |  |  |  | | |  | |  |  |  |  |
| **Own a vaping device^1^** |  |  | |  | | |  |  | | |  |  |  | | |  | |  |  |  |  |
| No |  | 326 (28.43) | | 659 (71.57) | | |  | 1.00 | | |  |  | 1.00 | | |  | |  |  |  |  |
| Yes |  | 93 (34.98) | | 243 (65.02) | | |  | 0.74 (0.40-1.37) | | | .336 |  | 0.84 (0.47-1.52) | | | .570 | |  |  |  |  |
| Don’t know/refused |  | 15 (40.48) | | 19 (59.52) | | |  | 0.58 (0.15-2.25) | | | .434 |  | 0.51 (0.14-1.92) | | | .319 | |  |  |  |  |
|  |  |  | |  | | |  |  | | |  |  |  | | |  | |  |  |  |  |
| **Last time vaped^2^** |  |  | |  | | |  |  | | |  |  |  | | |  | |  |  |  |  |
| Not in last 7 days but sometime in last 30 days |  | 138 (35.14) | | 264 (64.86) | | |  | 1.00 | | |  |  | 1.00 | | |  | |  |  |  |  |
| Not today but sometime in last 7 days |  | 112 (36.79) | | 242 (63.21) | | |  | 0.93 (0.49-1.77) | | | .828 |  | 1.07 (0.56-2.07) | | | .833 | |  |  |  |  |
| Earlier today |  | 184 (30.36) | | 415 (69.64) | | |  | 1.24 (0.69-2.25) | | | .473 |  | 1.68 (0.89-3.18) | | | .109 | |  |  |  |  |
|  |  | |  | |  | | |  | |  |  | | |  |  |  | | |  |  |  |
| **Urges to vape^3^** |  |  | |  | | |  |  | | |  |  |  | | |  | |  |  |  |  |
| Never |  | 66 (40.72) | | 160 (59.28) | | |  | 1.00 | | |  |  | 1.00 | | |  | |  |  |  |  |
| Several times a day |  | 114 (36.86) | | 250 (63.14) | | |  | 1.18 (0.51-2.72) | | | .702 |  | 1.64 (0.67-4.03) | | | .276 | |  |  |  |  |
| Every day or most days |  | 108 (26.78) | | 209 (73.22) | | |  | 1.88 (0.83-4.24) | | | .129 |  | 2.34 (0.96-5.74) | | | .063 | |  |  |  |  |
| At least once a week |  | 88 (32.41) | | 195 (67.59) | | |  | 1.43 (0.62-3.30) | | | .399 |  | 1.99 (0.84-4.69) | | | .117 | |  |  |  |  |
| Less than once a week |  | 51 (36.24) | | 101 (63.76) | | |  | 1.21 (0.45-3.22) | | | .704 |  | 1.51 (0.58-3.91) | | | .397 | |  |  |  |  |
| Don't know |  | 7 (41.21) | | 6 (58.79) | | |  | 0.98 (0.13-7.19) | | | .984 |  | 1.29 (0.22-7.45) | | | .772 | |  |  |  |  |
|  |  | |  | |  | | |  | |  |  | | |  |  |  | | |  |  |  |
| **Time to first vape^4^** |  |  | |  | | |  |  | | |  |  |  | | |  | |  |  |  |  |
| Within 5 minutes |  | 77 (40.75) | | 152 (59.25) | | |  | 1.00 | | |  |  | 1.00 | | |  | |  |  |  |  |
| 6-30 minutes |  | 80 (29.9) | | 189 (70.1) | | |  | 1.61 (0.69-3.77) | | | .271 |  | 1.59 (0.65-3.87) | | | .306 | |  |  |  |  |
| 31-60 minutes |  | 71 (28.41) | | 132 (71.59) | | |  | 1.73 (0.74-4.07) | | | .206 |  | 1.41 (0.61-3.23) | | | .420 | |  |  |  |  |
| 1-4 hours (i.e., in the morning) |  | 92 (40.02) | | 152 (59.98) | | |  | 1.03 (0.44-2.40) | | | .944 |  | 0.80 (0.36-1.76) | | | .572 | |  |  |  |  |
| 5-8 hours (i.e., in the afternoon) |  | 50 (24.27) | | 168 (75.73) | | |  | 2.15 (0.87-5.31) | | | .099 |  | 1.53 (0.64-3.63) | | | .339 | |  |  |  |  |
| More than 8 hours |  | 37 (46.67) | | 81 (53.33) | | |  | 0.79 (0.28-2.25) | | | .653 |  | 0.57 (0.21-1.54) | | | .268 | |  |  |  |  |
| Don't know/refused |  | 27 (41.93) | | 47 (58.07) | | |  | 0.95 (0.30-3.04) | | | .935 |  | 0.88 (0.30-2.60) | | | .820 | |  |  |  |  |
|  | |  | | |  |  | | |  | |  |  | | | |  |  |  | | |  |
| **E-Cigarette Dependence Scale (mean(95% CI))^5^** |  | 10.8 (9.9-11.7) | | 10.1 (9.7-10.6) | | |  | 0.96 (0.90-1.02) | | | .195 |  | 0.97 (0.91-1.04) | | | .464 | |  |  |  |  |

All data are weighted, except n which are unweighted. ^1^ Adjusted logistic regression models include age group, sex, race/ethnicity, current or returning student, perceived financial status, smoking status, and own a vaping device. ^2^ Adjusted logistic regression model includes last time vaped, age group, sex, race/ethnicity, current or returning student, perceived financial status, smoking status, and own a vaping device. ^3^ Adjusted logistic regression model includes urges to vape, age group, sex, race/ethnicity, current or returning student, perceived financial status, smoking status, and own a vaping device. ^4^ Adjusted logistic regression model includes time to first vape, age group, sex, race/ethnicity, current or returning student, perceived financial status, smoking status, and own a vaping device. ^5^ Adjusted logistic regression model includes E-Cigarette Dependence Scale, age group, sex, race/ethnicity, current or returning student, perceived financial status, smoking status, and own a vaping device.

| **Table A3. Reasons for brand choice among youth and young adults who have vaped in the past 30 days among those who used disposables vs. otherwise in England, 2022. Reasons are not mutually exclusive. All data except N are weighted. N = 1,355.** | | | | | | | | | | |
| --- | --- | --- | --- | --- | --- | --- | --- | --- | --- | --- |
|  | **N (%)** | |  | | **Use disposables (vs. otherwise)** | | | | | |
|  | **Responded otherwise**  **(N=434, 33.75%)** | **Use disposables most often**  **(N=921, 66.25%)** | |  | | **Unadjusted** | |  | **Adjusted** | |
|  |  |  |  |  |  | **OR (95% CI)** | **p** |  | **AOR (95% CI)^1^** | **p** |
| Better flavour / taste | 144 (27.49) | 432 (41.05) | |  | | **1.84 (1.12-3.02)** | **.017** |  | **1.84 (1.11-3.03)** | **.017** |
| Less expensive | 135 (22.34) | 309 (24.50) | |  | | 1.13 (0.70-1.82) | .622 |  | 1.36 (0.83-2.24) | .220 |
| Easier to get | 97 (23.68) | 265 (23.90) | |  | | 1.01 (0.60-1.71) | .964 |  | 1.00 (0.59-1.69) | .990 |
| Smoother to inhale | 93 (19.61) | 241 (21.76) | |  | | 1.14 (0.61-2.14) | .683 |  | 1.09 (0.56-2.11) | .798 |
| More popular among friends | 107 (22.32) | 280 (17.76) | |  | | 0.75 (0.43-1.31) | .316 |  | 0.71 (0.40-1.27) | .248 |
| Easier to use | 110 (25.67) | 217 (23.19) | |  | | 0.87 (0.50-1.52) | .634 |  | 0.91 (0.51-1.61) | .735 |
| Better looking | 82 (20.67) | 148 (19.09) | |  | | 0.91 (0.48-1.69) | .757 |  | 0.84 (0.45-1.58) | .592 |
| I was offered it | 51 (14.59) | 144 (13.82) | |  | | 0.94 (0.47-1.88) | .857 |  | 0.87 (0.45-1.67) | .669 |
| Stronger nicotine hit | 47 (14.78) | 128 (10.69) | |  | | 0.69 (0.34-1.40) | .306 |  | 0.67 (0.32-1.42) | .294 |
| Easier to hide | 46 (12.28) | 107 (11.06) | |  | | 0.89 (0.43-1.83) | .748 |  | 0.79 (0.39-1.59) | .510 |
| More fun | 57 (12.98) | 103 (15.92) | |  | | 1.27 (0.65-2.47) | .481 |  | 1.16 (0.59-2.28) | .674 |
| Less harmful | 49 (12.08) | 90 (14.20) | |  | | 1.20 (0.58-2.49) | .616 |  | 1.09 (0.52-2.31) | .814 |
| Better for quitting smoking | 66 (14.5) | 92 (12.69) | |  | | 0.86 (0.43-1.72) | .665 |  | 0.84 (0.37-1.90) | .672 |
| Don’t know/refused | 14 (2.03) | 13 (0.38) | |  | | **0.19 (0.06-0.62)** | **.006** |  | **0.11 (0.03-0.43)** | **.001** |

All data are weighted, except n which are unweighted. ^1^ Adjusted logistic regression models include age group, sex, race/ethnicity, current or returning student, perceived financial status, and smoking status.
